# Hardships of the Middle Class

**Published:** 1920-07-10

**Authors:** 


### THE PUBLIC WELL-BEING.

### Hardships of the Middle Class.

### Question Raised at the B.M.A.

The hard case of the middle class in the matter of medical treatment was mentioned by Sir Wilmot Herringham at one of the meetings of the British Medical Association last week. It is only one of the many respects in which the middle class is suffering in these days. Before the war it rarely had much of a margin for special contingencies, and since the war it has been the prey of the profiteer on one hand, and the greater sufferer on the other hand from the demands of labour for better times. Its earnings have been nearer the stationary mark than those of other classes, and it has had very little extra to enable it to meet its new liabilities. It is just above the line up to which the State provides education, medical treatment, food and various other things for nothing or next to nothing—and for which it helps to pay—and it has many obligations which well-paid labour does not have to meet. Some little deference was made to it in the framing of the new income tax scales, but the effect of that is not very great. It is good, however, to find that the British Medical Association has not overlooked it. Sir Wilmot Herringham said the two classes receiving the best treatment to be found in the country are the very rich and the very poor. The former can afford to pay not only for every requisite but for every comfort during illness. The latter are treated in hospitals, and are probably the better served of the two, but the middle class come very badly off. Their means do not allow of their paying large fees, yet are too large to allow them to be objects of charity. They are not taken into the hospitals, yet they cannot afford the cost of a nursing home, of an operation, or of the numerous accessory observations which both the physician and the surgeon now require. The only sound remedy, he believed, would be to extend to private patients the benefit and convenience of treatment at a hospital. Paying hospitals were one of the greatest needs of the time, and already existed in various forms. After sketching the ideal organisation of such a hospital, he said the scale of fees ought not to be a flat rate charged to all, but the rich should be made to pay more and the poor less.

Some such plan as this is the only hope and it is distinct from the more general proposals now being debated and in some cases actually in force for contributions from all patients in general hospitals. Dr. Addison received at the Ministry of Health recently a deputation from the Workhouse Nursing Association, consisting of Lady Edward Spencer Churchill, Miss James, Dr. Morley Fletcher, Mr. Dixon Kimber, hon. treasurer, and Mrs. Latter, hon. secretary. Dr. Fletcher explained the details of a scheme for the relief in sickness and old age of a large number of the semi-professional and educated classes whom circumstances arising out of the war had reduced to poverty. It was suggested that the excellent provision made in Poor-law infirmaries for chronic and incurable disease might be so extended and modified as to include, and be acceptable to, the new class of poor. Dr. Addison expressed sympathy with the proposal. This refers rather to the hardest cases of the new poor—in fact, to the new poorest—but it is part of the same problem, and in any national health scheme for which they will have to pay their share, the new poor are entitled to expect some consideration of their special circumstances.

